# Successful Resuscitation and Management of Cardiac Arrest in Pregnancy Due to Undiagnosed Severe Mitral Stenosis: A Case Report

**DOI:** 10.7759/cureus.35036

**Published:** 2023-02-15

**Authors:** Faraz Shafiq, Haris Sheikh

**Affiliations:** 1 Anaesthesiology, Aga Khan University Hospital, Karachi, PAK

**Keywords:** cardiac arrest in pregnancy, perimortem cesarean section, cardiopulmonary resuscitation, mitral stenosis, sudden cardiac arrest

## Abstract

Maternal collapse is a rare, potentially fatal event with associated feto-maternal morbidity and mortality. We report a case of severe mitral stenosis without any prior symptoms, that presented as acute cardiac decompensation leading to maternal collapse and cardiac arrest. A 35-year-old female in her 28th week of gestation presented to the emergency room with a four-hour history of per-vaginal discharge and leaking of amniotic fluid. Her past history, physical examination, and laboratory workup were unremarkable. An initial diagnosis of pre-term premature rupture of membranes (PPROM) was made and she was managed conservatively. Within four hours of the presentation, she developed shortness of breath, which gradually worsened, and the anesthesia team was requested to assess the patient. Upon arrival, the patient was in severe respiratory distress. She collapsed soon after and started frothing copiously from the mouth. Pulse was absent and cardio-pulmonary resuscitation (CPR) commenced. Endotracheal intubation was performed and the obstetric team was asked to prepare for a perimortem cesarean section, which was completed four minutes after the commencement of CPR and the baby was delivered alive and well with an APGAR score of 7 and 8 at one minute and five minutes of birth, respectively, and birth weight of 1.1 kg. CPR continued for 16 minutes after which a return of spontaneous circulation was achieved. Due to the unavailability of an ICU bed, the patient was shifted to OR where she stayed for the next five hours for further resuscitation. After a two-month-long ICU course, the patient was discharged in stable condition; her baby was discharged after a month of hospital stay. The expertise of anesthesiologists as resuscitators and peri-operative physicians helped in successful resuscitation, saving not just one but two lives in the process.

## Introduction

Maternal collapse is a rare, potentially fatal event with associated feto-maternal morbidity and mortality [1]. Management of maternal collapse is often complicated due to physiologic changes in pregnancy. Hence, maternity healthcare providers should be familiar with the management of maternal collapse as adequate and timely intervention can prevent significant morbidity [2]. Common causes of maternal collapse include seizures and vasovagal syncope, which are often self-limiting. However, thromboembolism, cardiac diseases, and sepsis-associated maternal collapse are associated with serious complications and adverse events [1]. Cardiac arrest in pregnancy is rare; however, it is associated with significant mortality with a case fatality rate of 42% [2].

We report a case of severe mitral stenosis without any prior symptoms, that presented as acute cardiac decompensation leading to maternal collapse and cardiac arrest for the first time in the second trimester and the role of anesthesiologists in successful resuscitation, post-event management, and survival of the patient and her baby.

## Case presentation

Our patient was a 35-year female, Gravida 1, Para 0, at the 28th week of gestation with no associated comorbid condition. She had conceived after nine years of marriage and was booked at a local maternity facility at the district level. She presented to us in ER, with a four-hour history of vaginal discharge and leaking. Her past medical, surgical, and family histories were unremarkable. She had no history of prior hospitalization.

Upon physical examination, her blood pressure (BP) was 101/70 mmHg, oxygen saturation level was 98% on room air, pulse rate was 112 beats per minute and regular. General physical, respiratory, and cardiovascular examinations were unremarkable. Initial laboratory investigations including complete blood count (CBC), blood urea nitrogen (BUN), creatinine, electrolytes, and coagulation profile were within normal limits. Ultrasound pelvis with uterine artery doppler was also performed, which showed a single-alive uterine fetus corresponding to 28 weeks of gestation. The estimated weight was 1100 grams, placenta in the posterior upper segment, an open cervical os with fluid in the vagina, and a normal uterine artery doppler. An initial diagnosis of pre-term premature rupture of membranes (PPROM) was made, and it was decided to keep the patient under observation in the labor room suite and manage her conservatively.

Within four hours of presentation, the patient started developing shortness of breath (SOB), which initially resolved upon sitting up and supplemental oxygen via nasal prongs at 2 liters per minute. However, the SOB gradually worsened. The patient also started becoming hypertensive with multiple BP readings around 150/100 mmHg, for which labetalol infusion was started at 20 mg/hour. However, her SOB worsened, and she started developing tachycardia and tachypnea. Her oxygen therapy was gradually escalated to 10 liters per minute via face mask. Rapid Response Team was called to assess the patient urgently and an informal request was made to the anesthesia team of the labor operating room to assess the patient as well.

Upon arrival at the labor room suite, the anesthesia team saw the patient leaning forward, gasping for air, and in severe respiratory distress. Her BP was 180/109 mmHg, pulse rate was 156/min, and oxygen saturation levels of 40%. Within a few minutes, the patient collapsed and started frothing copiously from the mouth. Pulse was checked which was absent. The anesthesia team commenced cardiopulmonary resuscitation (CPR) after raising the code blue alarm and simultaneously alerted the primary team to prepare for perimortem cesarean section at the bedside. In the next two minutes, the patient developed V-Fib, defibrillation with 200J was done, and endotracheal intubation was performed. Perimortem cesarean section was performed four minutes after the commencement of CPR as per the Royal College of Obstetricians and Gynaecologists (RCOG) and American Heart Association (AHA) guidelines.

The baby was delivered alive and well in the next two minutes and shifted to the neonatal intensive care unit (ICU) for further management. The baby's initial weight was 1.1 kg and an APGAR of 7 and 8 at one minute and five minutes of birth, respectively. The patient developed V-fib three more times, for which defibrillation was done accordingly. CPR continued for 16 minutes led by the anesthesia team after which spontaneous circulation was achieved. A laboratory workup, chest x-ray, and portable echocardiogram were ordered, and the primary team was advised to shift the patient to the surgical ICU; however, there was full occupancy in the ICU. Since the patient required immediate ICU care, one of the labor room ORs was converted into a temporary ICU setting for further intensive care management. The patient stayed in the OR for the next five hours where the anesthesia team performed post-event resuscitation and management.

**Figure 1:**
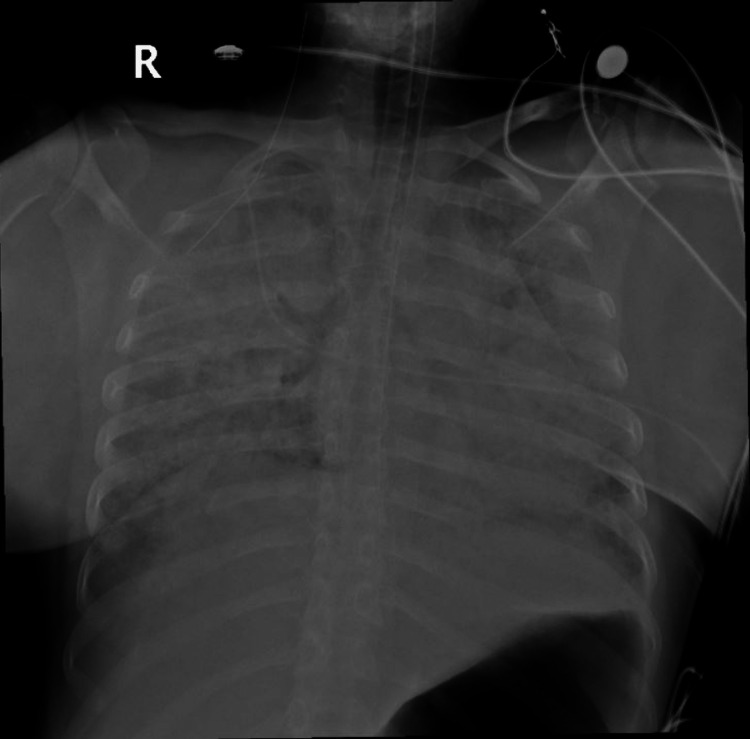
Chest x-ray (anteroposterior view) after intubation

During this time, the patient was sedated, relaxed, and mechanically ventilated via the anesthesia machine ventilator. Her peak airway pressures were high and ranged between 50-60 cmH20 and she required frequent endobronchial suctioning because of massive frothy secretions. An arterial line and a central line were placed. Her laboratory workup came back positive for acidosis and significant hypoxemia with a pH of 7.09 and pO2 of 50.7 on ABGs. Chest x-ray showed inhomogeneous opacification of bilateral lung fields (upper, middle, and lower zones) resulting in a complete white-out representing gross pulmonary edema (Figure 1).

**Figure 2:**
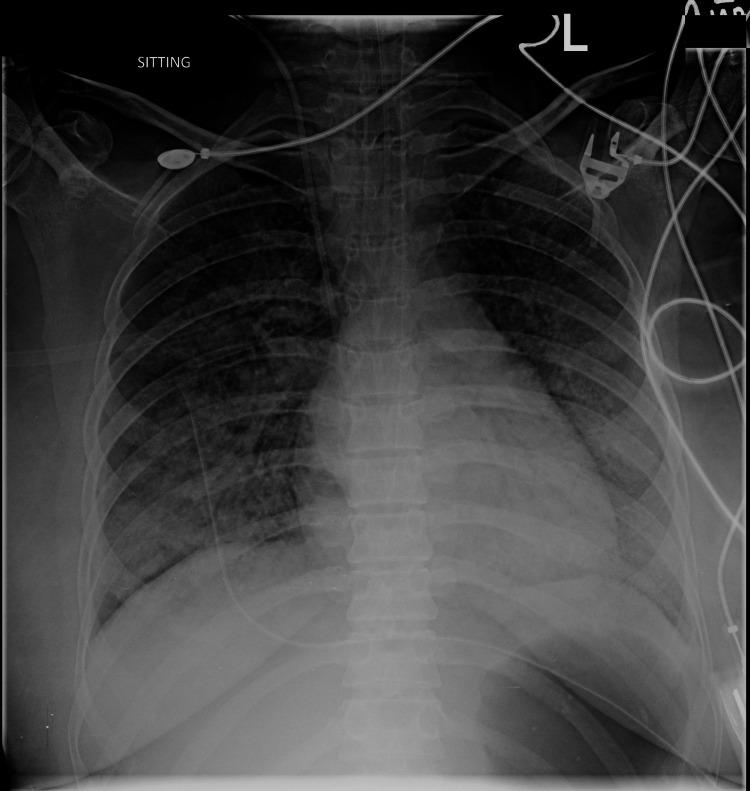
Chest x-ray (anteroposterior view) after resuscitation

Her portable echocardiogram showed a hockey stick appearance of the anterior leaflet and restricted mobility of the posterior leaflet causing severe mitral stenosis with a valve area of 1.4 cmSq, moderate pulmonary artery hypertension (PAH) with estimated pulmonary artery systolic of 45 mmHg and a preserved ejection fraction. The patient was given 40 mg of furosemide intravenously and she subsequently poured out 800 ml of urine over the next three hours. Empiric broad-spectrum intravenous antibiotics (meropenem and vancomycin) were started, and a single dose of intravenous hydrocortisone 100 mg was given. Her peak airway pressures reduced to 25-30 cmH20, blood pressures came back to lie within normal range, and she was shifted to ICU after five hours of resuscitation in the OR. Upon initial assessment, she was alert, responsive, and obeying commands. She was spontaneously opening her eyes (E4), and obeying commands for motor movements (M6) making a total Glasgow Coma Scale (GCS) of 10T. Her repeat chest x-ray showed interval resolution of pulmonary edema with mild inhomogeneous opacifications (Figure 2).

She stayed in the ICU for about two months due to certain complications, which primarily included otitis media, hospital-acquired pneumonia, acute kidney injury (AKI) with complete resolution, critical illness neuropathy, and failure to wean. She underwent percutaneous mitral commissurotomy for mitral stenosis after which her pulmonary edema gradually resolved and later underwent tracheostomy. She was discharged in stable condition. Her baby girl survived and was discharged after a month of hospital stay due to concerns of prematurity.

## Discussion

The RCOG has defined maternal collapse as an acute event involving the cardiorespiratory systems and/or brain, resulting in a reduced or absent conscious level (and potentially death), at any stage in pregnancy and up to six weeks after delivery [3]. This is a rare condition with an incidence between 0.14 and 6 per 1000 births [4]. Vasovagal syncope and seizures are the most common causes of maternal collapse. Most of these incidents are self-limiting with a favorable outcome. Other causes of maternal collapse include obstetric hemorrhage, thromboembolism, amniotic fluid embolism, hemorrhage of non-obstetric origin, cerebral hemorrhage or infarction, cerebral venous sinus thrombosis, and metabolic or anesthetic causes [1]. Maternal cardiac arrest occurs in approximately 2.78 per 100,000 maternities and the mortality rate reaches 42% in the United Kingdom. In the United States, the incidence of cardiac arrest is 8.5 per 100,000 admissions for delivery [3]. Among cardiac causes, acute myocardial infarction, aortic dissection, congenital heart diseases, and cardiomyopathy are the frequently encountered etiologies [2].

Rheumatic heart disease is responsible for the majority of cardiac cases and mitral stenosis is the most frequently observed valvular lesion in pregnant females with cardiac disease in developing countries such as Pakistan [5]. Symptoms of valvular heart disease include SOB, fatigability, syncope, and palpitations. Associated signs may include clubbing, raised jugular venous pulsation (JVP), cyanosis, cardiomegaly, new murmurs, fine crepitations on auscultation, and arrhythmia. These signs and symptoms are often exacerbated during pregnancy due to associated cardiovascular changes [5]. However, a previously asymptomatic patient of mitral stenosis, which first presents as the maternal collapse, is a potentially rare presentation.

Cardiac decompensation and pulmonary edema may occur in pregnancy with previously asymptomatic mitral stenosis during the second or third trimester. This is in line with the disease/event course of our patient. Patients with asymptomatic valvular insufficiency tend to tolerate volumetric overload during pregnancy much better [6]. Maternal mortality is highest during labor and immediately after delivery. The incidence of maternal cardiac complications correlates with the severity of the mitral stenosis (67% for severe, 38% for moderate, and 26% for mild disease). Mortality rates for New York Heart Association (NYHA) classes I and II amount to <1%, whereas classes III and IV, have a mortality rate between 5 and 15%. The perinatal mortality rate for classes III and IV is as high as 20-30% [7].

The role of our anesthesia team was particularly important in this case as the presence of anesthesiologists in the room helped recognize the event minutes before it happened, which in turn alerted all essential personnel to respond immediately to save not just the mother but her baby as well. Furthermore, the expertise of anesthesiologists as resuscitators and peri-operative physicians helped in successful resuscitation during and immediately after the event, saving not just one but two lives in the process.

## Conclusions

With the incidence of mitral stenosis being the highest among the valvular cardiac disease in pregnancy, it should always be considered as a differential when dealing with maternal collapse or cardiac arrest in pregnancy. Adequate training of healthcare workers in the identification of symptoms and signs of cardiac disease for early detection and management is crucial. Furthermore, healthcare workers should be well-versed in resuscitation protocols during pregnancy for better outcomes. We recommend cardiovascular examination with emphasis on auscultation for heart sounds and murmurs, observation for raised JVP, and early echocardiography to be performed as routine in maternal collapse, as in this patient for early diagnosis and management, which may lead to a more favorable prognosis.
